# On the origin of amphi-enterobactin fragments produced by *Vibrio campbellii* species

**DOI:** 10.1007/s00775-022-01949-0

**Published:** 2022-07-14

**Authors:** Aneta M. Jelowicki, Alison Butler

**Affiliations:** Department of Chemistry and Biochemistry, University of California, Santa Barbara, CA 93106-9510 USA

**Keywords:** Iron, Siderophore, Biosynthesis, Hydrolysis, Genomics, Catechol

## Abstract

Amphi-enterobactin is an amphiphilic siderophore isolated from a variety of microbial *Vibrio* species. Like enterobactin, amphi-enterobactin is a triscatecholate siderophore; however, it is framed on an expanded tetralactone core comprised of four l-Ser residues, of which one l-Ser is appended by a fatty acid and the remaining l-Ser residues are appended by 2,3-dihydroxybenzoate (DHB). Fragments of amphi-enterobactin composed of 2-Ser-1-DHB-FA and 3-Ser-2-DHB-FA have been identified in the supernatant of *Vibrio campbellii* species. The origin of these fragments has not been determined, although two distinct isomers could exist for 2-Ser-1-DHB-FA and three distinct isomers could exist for 3-Ser-2-DHB-FA. The fragments of amphi-enterobactin could originate from hydrolysis of the amphi-enterobactin macrolactone, or from premature release due to an inefficient biosynthetic pathway. Unique masses in the tandem MS analysis establish that certain fragments isolated from the culture supernatant must originate from hydrolysis of the amphi-enterobactin macrolactone, while others cannot be distinguished from premature release during biosynthesis or hydrolysis of amphi-enterobactin.

**Graphical abstract:**

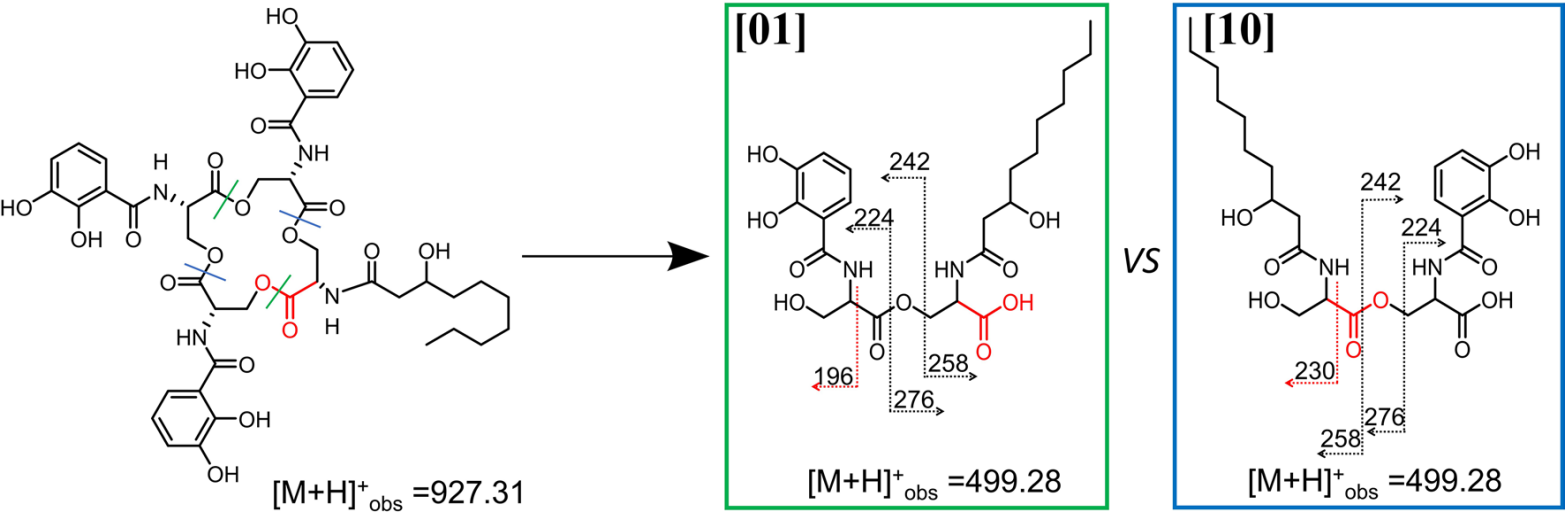

**Supplementary Information:**

The online version contains supplementary material available at 10.1007/s00775-022-01949-0.

## Introduction

Iron is a cofactor required by many enzymes involved in essential cellular processes. However, obtaining iron becomes challenging due to the low solubility of iron (III). One strategy that bacteria have evolved to obtain iron is the biosynthesis of siderophores, low molecular weight organic compounds that bind Fe(III) with high affinity. These Fe(III)-siderophore complexes are taken up by the cell through outer membrane receptor proteins.

**Fig. 1:**
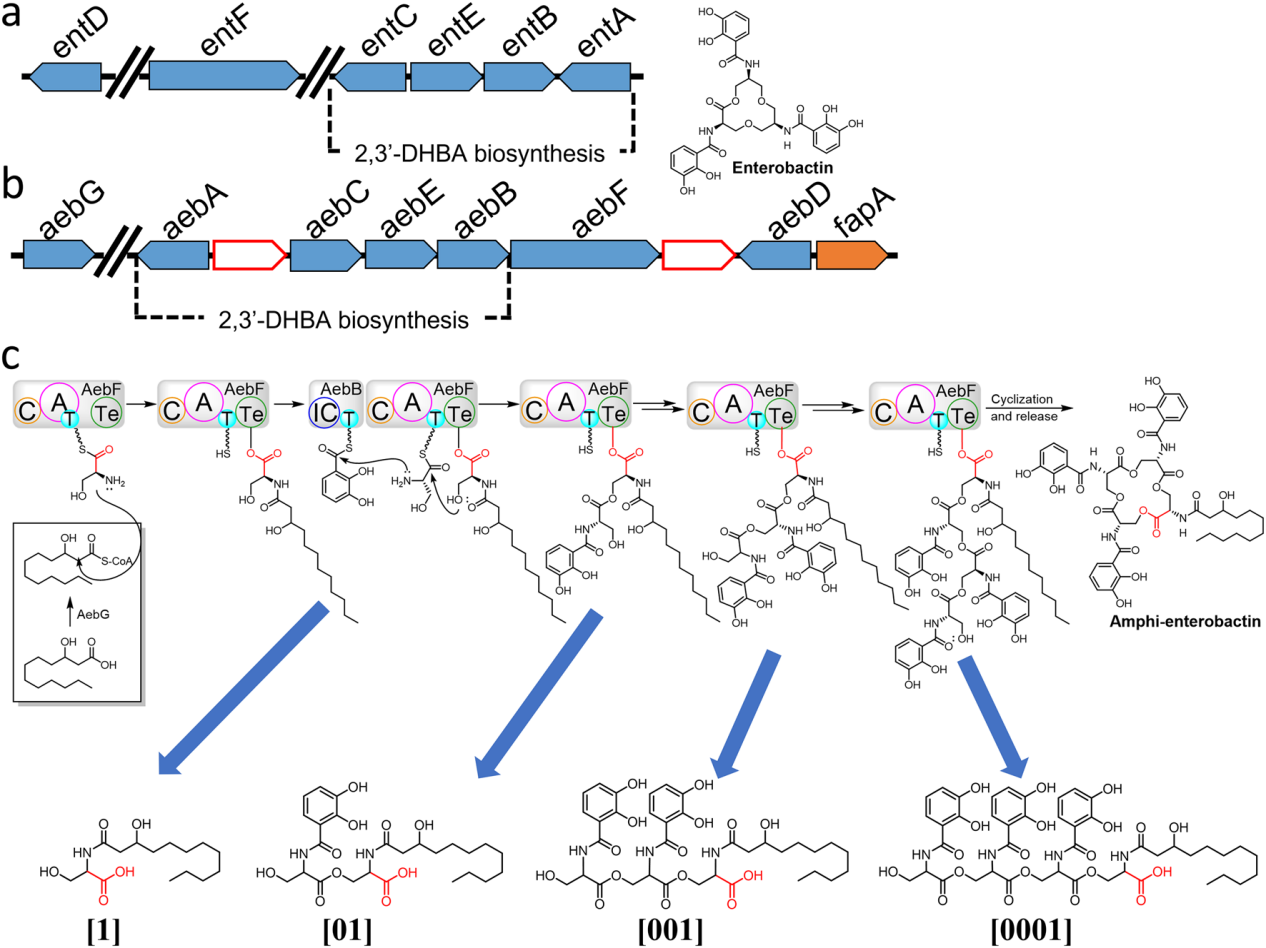
Biosynthesis of amphi-enterobactin. **a** The *entABCDEF* biosynthetic gene cluster. **b** The *aebABCDEF* biosynthetic gene cluster first identified in *Vibrio harveyi* BAA-1116 [1]. Genes involved in siderophore biosynthesis and transport are represented by blue and orange arrows, respectively. White arrows represent hypothetical proteins whose function has not yet been determined. **c** Biosynthesis of amphi-enterobactin catalyzed by NRPS AebF. The potential points of pre-release of fragments in the biosynthesis of amphi-enterobactin are indicated (blue arrows). Each potential early release product has the fatty acid appended to the amine of a C-terminal l-Ser. *C* condensation domain; *A* adenylation domain; *T* thiolation domain; *TE* thioesterase domain

Amphi-enterobactin (Fig. 1) [1] was initially isolated from *Vibrio campbellii* ATCC BAA-1116 (formerly *V. harveyi* BAA-1116), a model bacterium for quorum sensing because of its quorum-regulated bioluminescence [2]. Enterobactin, utilized by many bacterial species, is a macrolactone of *tris*-(*N*-2,3-dihydroxybenzoyl-l-serine) that coordinates iron(III) with three 2,3-dihydroxybenzoyl (DHB) catechol groups. Amphi-enterobactin is a triscatecholate siderophore resembling enterobactin, although distinguished by an expanded tetralactone core, and decorated by a fatty acid appended at the amine of the additional l-Ser [1]. Multiple strains of *V. campbellii* and *V. harveyi* have been shown to produce a suite of amphi-enterobactins with varying fatty acyl groups [1, 3]. These fatty acid appendages can range in length (C10–C16), degree of unsaturation, and hydroxylation [1, 3, 4]. *V. campbellii* CAIM 519T produced the full suite (C10–C16) amphi-enterobactins in greater amounts than *V. campbellii* BAA-1116 [3].

*V. campbellii* BAA-1116 contains a set of genes homologous to the biosynthetic gene cluster (BGC) of enterobactin, *entA-F* (Fig. 1A), yet instead of enterobactin, the strain produces amphi-enterobactin (Fig. 1B, C) [1]. In addition to the amphi-enterobactin *aebA-F genes*, the gene *aebG* encoding a long-chain fatty acid Co-A ligase (FACL) is located nearby this BGC [1]. The biosynthesis of 2,3-dihydroxybenzoic acid (2,3-DHBA) is carried out by AebABCE. Zane et al*.* [1] established that the biosynthesis of amphi-enterobactin begins by appending an AebG-activated fatty acid to l-Ser loaded on AebF (Fig. 1C). FACL enzymes are known to activate fatty acids to fatty acyl-CoA thioesters before integrating with the nonribosomal peptides [5, 6]. Thus, this FACL initiates the biosynthetic process of amphi-enterobactin by appending the FA to the first loaded l-Ser residue on AebF NRPS. AebF continues its bifunctional activity of catalyzing the formation of amide bonds between DHB and another l-Ser, respectively. The thioesterase domain of AebF ultimately catalyzes the release of amphi-enterobactin through intramolecular cyclization, generating the macrolactone and releasing amphi-enterobactin from the NRPS [1].

Several bacterial strains, *V. campbellii* BAA-1116, *Burkholderia cepacian* K56-2, and *V. vulnificus* MO6-24/O, have been shown to engage in quorum-sensing regulation of siderophore production, where high cell density leads to an accumulation of quorum sensing molecules, which with the Fe(II)–Fur complex decreases siderophore production [2, 4, 7, 8]. A recent study reported the presence of amphi-enterobactin-related soluble fragments, particularly 2,3-dihydroxybenzoic acid (DHBA) and 2,3-dihydroxybenzoyl-l-serine (DHB-Ser), along with linearized amphi-enterobactin fragments mass spectrometry [4]. DHBA and DHB-Ser were found to be more abundant in comparison with amphi-enterobactin. McRose et al. [4] propose two possible sources of DHBA and DHB-Ser: premature release from the biosynthetic pathway or degradation of amphi-enterobactins. Because of the accumulation of DHBA and DHB-Ser found in the supernatant of *V. campbellii* BAA-1116, the study suggested an inefficient amphi-enterobactin biosynthetic process [4].

Amphi-enterobactin hydrolysis products composed of two l-Ser residues, one 2,3-dihydroxybenzoate (2,3-DHB) group and a fatty acid, have been reported previously [1, 4]. In this report, we use a shorthand notation for these fragments, based on a binary code [9, 10], where the number [1] depicts l-Ser appended by the fatty acid, and **[0]** represents the l-Ser appended by DHB. In a 2-Ser-1-DHB-FA fragment where the fatty acid is appended to the C-terminal l-Ser, the binary code is **[01]**. If the fatty acid is appended to the N-terminal l-Ser, the binary code is **[10]**. The same designation is followed for 3-Ser-2-DHB-FA, where the fatty acid can be appended to the terminal l-Ser **[001]**, the internal l-Ser **[010]**, or the N-terminal l-Ser **[100]**. This binary nomenclature was originally used to describe the isomers of desferrioxamine B and was adapted here to denote the position of the FA [9, 10].

We have investigated the origin of the amphi-enterobactin fragments present in the culture supernatant of *V. campbellii* CAIM 519T in greater detail. Fragments associated with premature release during biosynthesis could only be **[01]**, **[001]**, and **[0001]**, where the fatty acid is appended to the C-terminal Ser. If premature release from the NRPS is the only source of the hydrolysis products, we would only see these three fragments. However, if hydrolysis of the fully formed amphi-enterobactin macrolactone occurs, a mixture of fragments will be observed, including **[10]**, **[100]**, and **[1000]**, which would have a unique tandem MS signature, described below, that would not be present in fragments **[01]**, **[001]**, and **[0001]**.

We report herein a mass fragmentation analysis that establishes these amphi-enterobactin hydrolysis fragments arise from the full siderophore, although we cannot rule out premature release. The amphi-enterobactin macrolactone siderophore is in fact produced as supported by the tandem MS analysis of the hydrolysis products.

## Materials and methods/experimental

### General experimental procedures

A Varian Cary-Bio 300 UV–visible spectrophotometer was used to monitor microbial growth at 600 nm. Analytical HPLC was used to analyze both the supernatant and cell pellet extracts from *V. campbellii* CAIM 519T to identify the production of both the amphi-enterobactin macrolactone and hydrolysis products. Mass spectrometry analysis was carried out on a Waters Xevo G2-XS QTof with positive-mode electrospray ionization coupled to an ACQUITY UPLC H-Class system with a Waters BEH C18 column.

### Cultivation of *Vibrio campbellii* CAIM 519T and siderophore isolation

*V. campbellii* CAIM 519T was cultured in low-iron artificial seawater medium containing casamino acids (10 g/L), NH_4_Cl (19 mM), Na_2_HPO_4_·7H_2_O (4.6 mM), MgSO_4_·7H_2_O (50 mM), CaCl_2_ (10 mM), trace metal grade NaCl (0.5 M), glycerol (41 mM), HEPES buffer (10 mM; pH 7.4), NaHCO_3_ (2 mM), biotin (8.2 μM), niacin (1.6 μM), thiamin (0.33 μM), 4-aminobenzoic acid (1.46 μM), pantothenic acid (0.21 μM), pyridoxine hydrochloride (5 μM), cyanocobalamin (0.07 μM), riboflavin (0.5 μM), and folic acid (0.5 μM). Two-liter cultures were grown in acid-washed 4-L Erlenmeyer flasks on an orbital shaker (180 rpm) at room temperature (OD_600_) while monitoring the growth until the culture reached stationary phase.

The cultures were harvested by centrifugation (6000 rpm, 30 min, 4 °C). The supernatant was decanted, and the cell pellet was resuspended in methanol (25 mL/pellet), transferred into 50-mL conical tubes, and shaken overnight at 180 rpm, 4 °C. The methanol extract was centrifuged (6000 rpm, 10 min, 4 °C), filtered through a 0.22-μm membrane, and concentrated under vacuum to one-third the original volume.

Both the supernatant and the cell pellet extracts were purified with XAD resin. The cell pellet extract was diluted with four times the volume with doubly deionized water (Milli-Q IQ). The supernatant and cell pellet extract were incubated with Amberlite XAD-2 resin for 4 h at 120 rpm, 25 °C. After 4 h, the XAD resin was washed with 2 L of doubly deionized water. From the cell pellet extract, siderophores were eluted with 90% methanol. From the supernatant, siderophores were eluted with 80% methanol. The eluent was concentrated under vacuum to dryness and dissolved in 5 mL of methanol.

### UPLC-MS and MS/MS analysis of extracts

Extracts were analyzed through positive ion mode ESI–MS on a Waters Xebo G2-XS QTof coupled to a Waters Acquity H-Class UPLC system. The extracts of the culture supernatant were analyzed with a linear gradient of 0–100% CH_3_CN (0.1% formic acid), while the cell pellet extracts were analyzed with a linear gradient of 50–100% CH_3_CN (0.1% formic acid) in ddH_2_O (0.1% formic acid) over 10 min. For MSMS analysis, a collision energy profile of 20, 25, 30 kEV was employed. Using MassLynx 4.1, chromatograms for masses of interest were generated and molecular ion peaks quantified by integration (ApexTrack algorithm).

## Results and interpretation

### Origin of the amphi-enterobactin fragments: premature release during biosynthesis or macrolactone ester hydrolysis

**Fig. 2:**
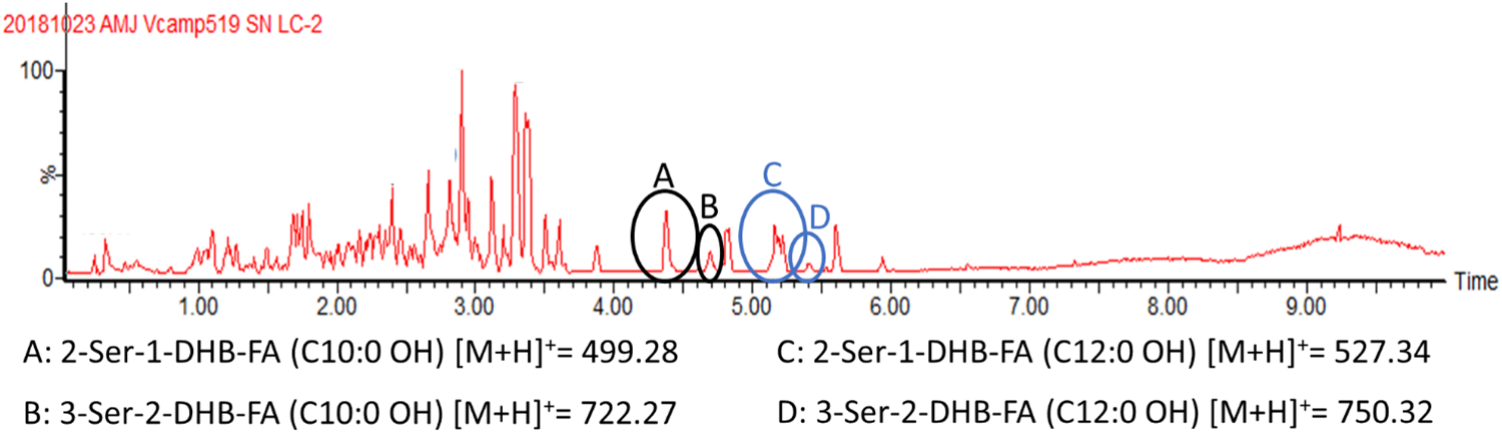
LC–MS of the *Vibrio campbellii* CAIM 519T supernatant. Peaks A–D correlate to masses of predicted amphi-enterobactin fragments

**Fig. 3:**
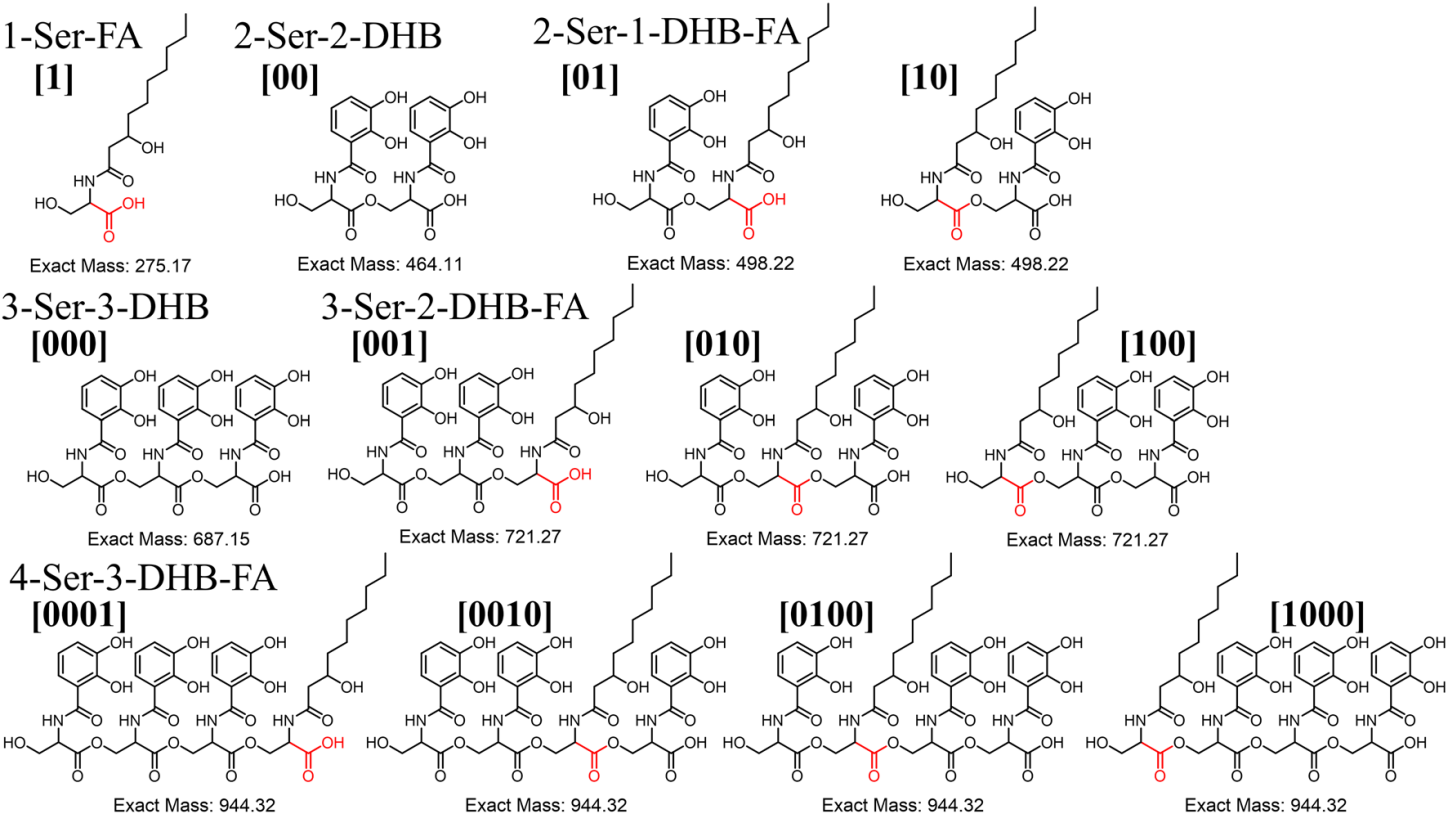
The possible hydrolysis fragments from amphi-enterobactin with a C10:0–OH fatty acid. Compounds **[01]**, **[001]**, **[0001**] are the only structural possibilities for premature release during biosynthesis. A mixture of the compounds shown here would suggest breakdown by an esterase. The carbonyl of ^L^Ser appended by the FA during biosynthesis is shown in red. This carboxyl would be tethered to the thioesterase domain during biosynthesis [1]

While it has been established that *Vibrio campbellii* CAIM 519T produces a suite of amphi-enterobactins, with fatty acids ranging from C_10_ to C_14,_ which are either saturated or monohydroxylated [3], fragments of these amphi-enterobactins are also present in the culture supernatant of *V. campbelli* CAIM 519T (Figs. 2, 3 and S2). We have turned to tandem MS to investigate whether selected fragments originate from hydrolysis of the amphi-enterobactin macrolactone siderophore.

The four circled peaks (A–D) in the UPLC chromatogram (Fig. 2) correlate with masses of amphi-enterobactin fragments identified in the supernatant. The species eluting at 4.3 min, labeled Peak A, reveals a protonated mass of *m/z* 499 [M + H]^+^, which matches the composition of the amphi-enterobactin fragment with a C10:0–OH fatty acid, referred to as 2-Ser-1-DHB-FA^C10:0−OH^. Peak C (*m/z* 527), eluting at 5.1 min, is analogous to Peak A although with a C12:0–OH fatty acid, i.e., 2-Ser-1-DHB-FA^C12:0−OH^. Two structural isomers are possible with each of these compositions, depending on the positions of the fatty acid and 2,3-DHB in reference to the serine ester backbone; the fatty acid may be appended to either the C-terminal l-Ser, depicted by the binary code **[01]**, or the N-terminal l-Ser, depicted by **[10]** (Fig. 3).

The species eluting at 4.6 min and 5.4 min are associated with Peak B and Peak D, respectively (Fig. 2). Peak B reveals a protonated molecular mass of *m/z* 722 [M + H]^+^, consistent with the composition 3-Ser-2-DHB-FA^C10:0−OH^, and peak D (*m/z* 750) is associated with the equivalent C12:0–OH fatty acid derivative. Three structural isomers exist for 3-Ser-2-DHB-FA (Fig. 3) in which the fatty acid may be appended to the C-terminal l-Ser **[001]**, the internal l-Ser **[010]**, or the N-terminal l-Ser **[100]**. The structural variability of isomers **[001]**, **[010]** and **[100]** prompted further considerations for the origin of these fragments.

The protonated molecular masses at *m/z* 945.33 [M + H]^+^ and *m/z* 973.35 [M + H]^+^ would be consistent with production of the 4-Ser-3-DHB-FA isomers for the C10:0–OH and C12:0–OH fatty acids, respectively. Four potential isomers could be formed, i.e., **[0001]**, **[0010]**, **[0100]**, and **[1000]**; however, due to the trace quantity produced, tandem MS characterization was not carried out. The complete set of isomers along with the associated binary nomenclature is shown in Fig. 3.

Biosynthesis of amphi-enterobactin is initiated during fatty acyl-CoA thioester acylation of l-Ser-S-P-pant-AebF [1]. Thus, the carboxyl group interacting with the thioesterase domain throughout the amphi-enterobactin biosynthesis will always be appended to the fatty acid that was loaded onto l-Ser. Premature release of amphi-enterobactin fragments along the biosynthetic pathway could potentially occur at the thioesterase domain of the NRPS, releasing a fragment with the fatty acid appended to the C-terminal Ser, as in **[01]**, **[001]**, or **[0001]** (Fig. 1).

Structural variation within fragments increases if hydrolysis products arise from the fully formed amphi-enterobactin macrolactone. While this set of fragments may contain the fatty acid appended to the C-terminal Ser, as in the premature release fragments **[01]**, **[001]**, or **[0001]**, other fragments with the fatty acid appended at each of the other Ser residues in the oligoserine backbone may be formed as well. Depending on the site of macrolactone hydrolysis, all of the structures in Fig. 3 may be considered hydrolysis products from amphi-enterobactin.

### Structural differentiation among the 2-Ser-1-DHB-FA fragments of amphi-enterobactin

**Fig. 4:**
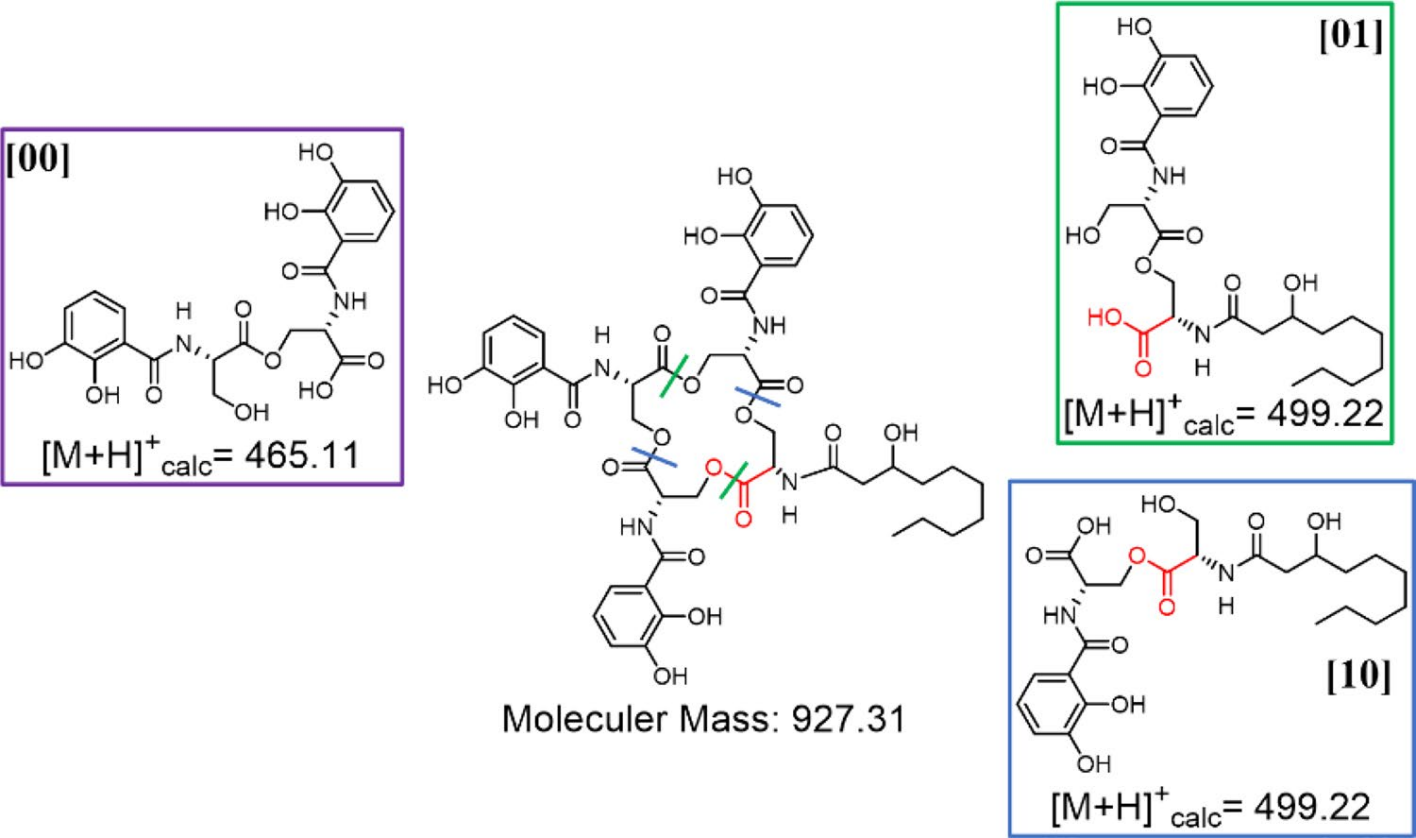
The possible di-SerC10:0–OH fragments that could arise from ester hydrolysis of amphi-enterobactin. Esters hydrolyzed directly opposite one another form the di-Ser fragments, **[00]**, **[01]**, and **[10]**

**Fig. 5:**
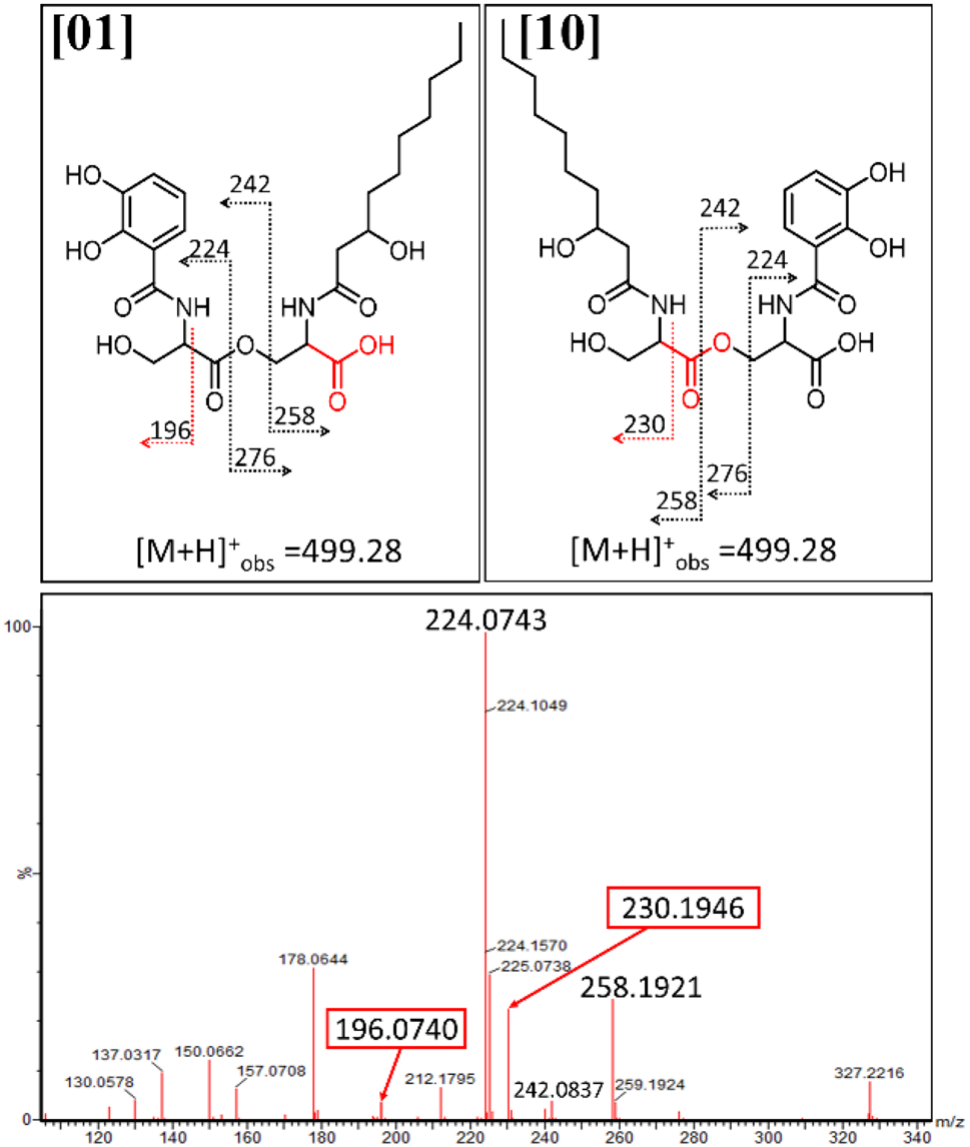
MS–MS of *m*/*z* 499.28 for differentiation between **[01]** and **[10].** A fragment ion of *m/z* 196 is consistent with premature release, while a fragment ion of *m/z* 230 is consistent with the hydrolysis of the amphi-enterobactin macrolactone

Three distinct di-Ser hydrolysis fragments can be formed from dual ester hydrolysis of the amphi-enterobactin macrolactone, only two of which would have a 2-Ser-1-DHB-FA motif, **[10]** and **[01]** (Figs. 4 and S1 for the C10:0–OH and C12:0–OH fatty acids, respectively). The third hydrolysis fragment would lack the fatty acid as 2-Ser-2-DHB, **[00]** (Fig. 4). Tandem mass spectrometry analysis can be used to differentiate between structures **[01]** and **[10]** based on unique MS/MS signature fragments (Figs. 5 and S3). Focusing first on the C10:0–OH derivatives, the fragment with a protonated mass of *m/z* 196 [M + H]^+^ is specific to **[01]**, while structure **[10]** would have a fragment with a protonated mass of *m/z* 230 [M + H]^+^.

The ESI–MS/MS spectrum of the product with a protonated mass of *m/z* value 499.28 [M + H]^+^ (**2-Ser-1-DHB-FA**^**C10:0−OH**^) shows fragments at both *m/z* 196 and *m/z* 230 (Fig. 5). The same pattern is observed for **2-Ser-1-DHB-FA**^**C12:0−OH**^ although with analogous fragments at *m/z* 196 and *m/z* 258 (Fig. S3). The mixture of both unique fragments is evidence that the amphi-enterobactin macrolactone is produced and is hydrolyzed to **[01]** and **[10]**, although the presence of both **[01]** and **[10]** does not rule out premature release during the biosynthesis as the origin of some **[01]**.

### Structural differentiation among the 3-Ser-2-DHB-FA fragments of amphi-enterobactin

**Fig. 6:**
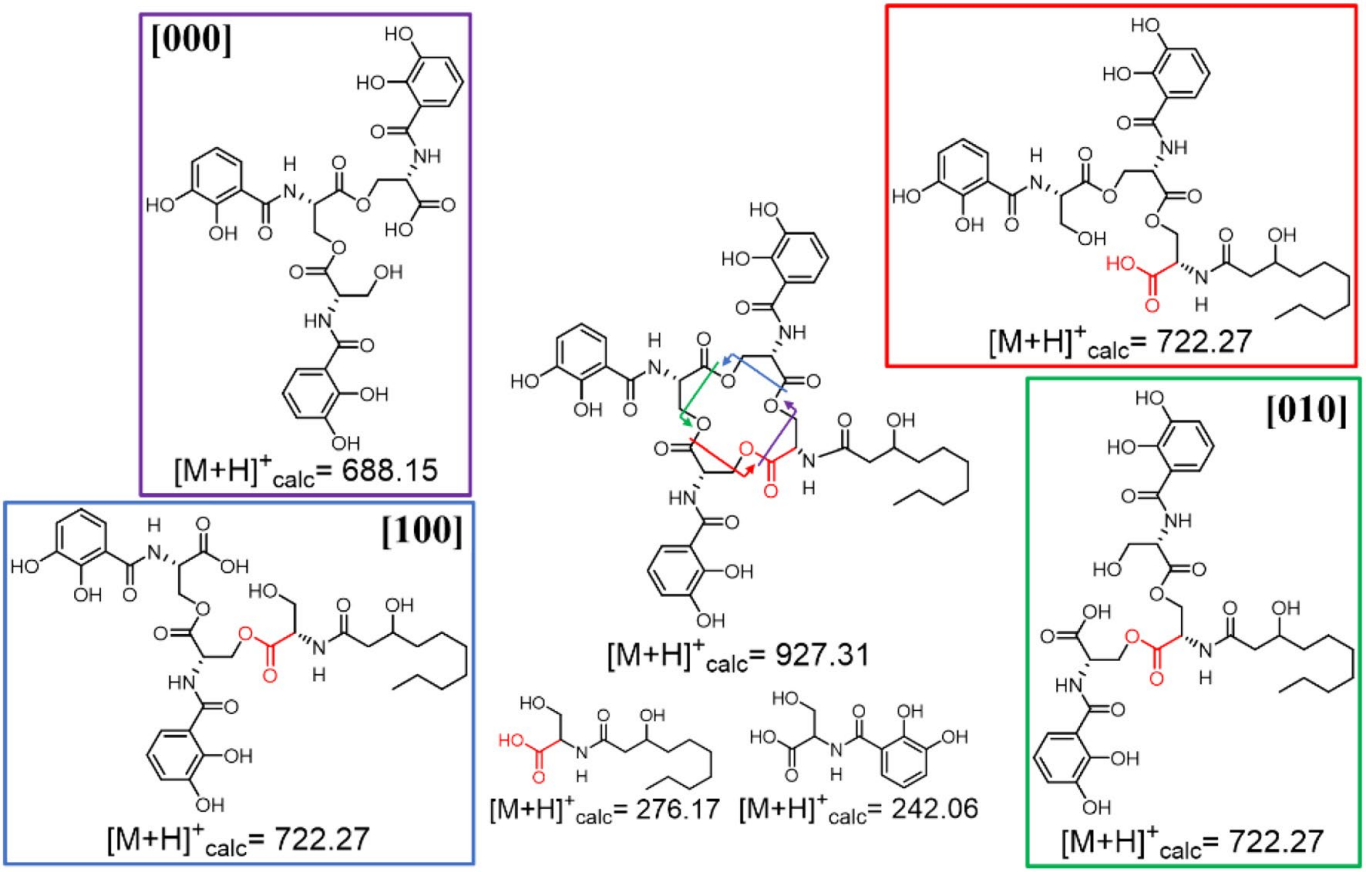
The possible tri-Ser^C10:0−OH^ fragments that could arise from ester hydrolysis of amphi-enterobactin. Hydrolysis at two adjacent esters within the amphi-enterobactin macrolactone would form the tri-Ser fragments, **[000]**, **[001]**, **[010]** and **[100]**

Along with **2-Ser-1-DHB-FA**, **3-Ser-2-DHB-FA** compounds were also observed. Four distinct tri-Ser hydrolysis fragments could be formed from dual hydrolysis of adjacent esters within the amphi-enterobactin macrolactone, only three of which would have a 3-Ser-2-DHB-FA motif, **[100]**, **[010],** and **[001]** (Figs. 6 and S1 for the C10:0–OH and C12:0–OH fatty acids, respectively). The fourth hydrolysis fragment would lack the fatty acid, with 3-Ser-3-DHB, **[000]** (Fig. 6), which is the equivalent of linear enterobactin.

**Fig. 7:**
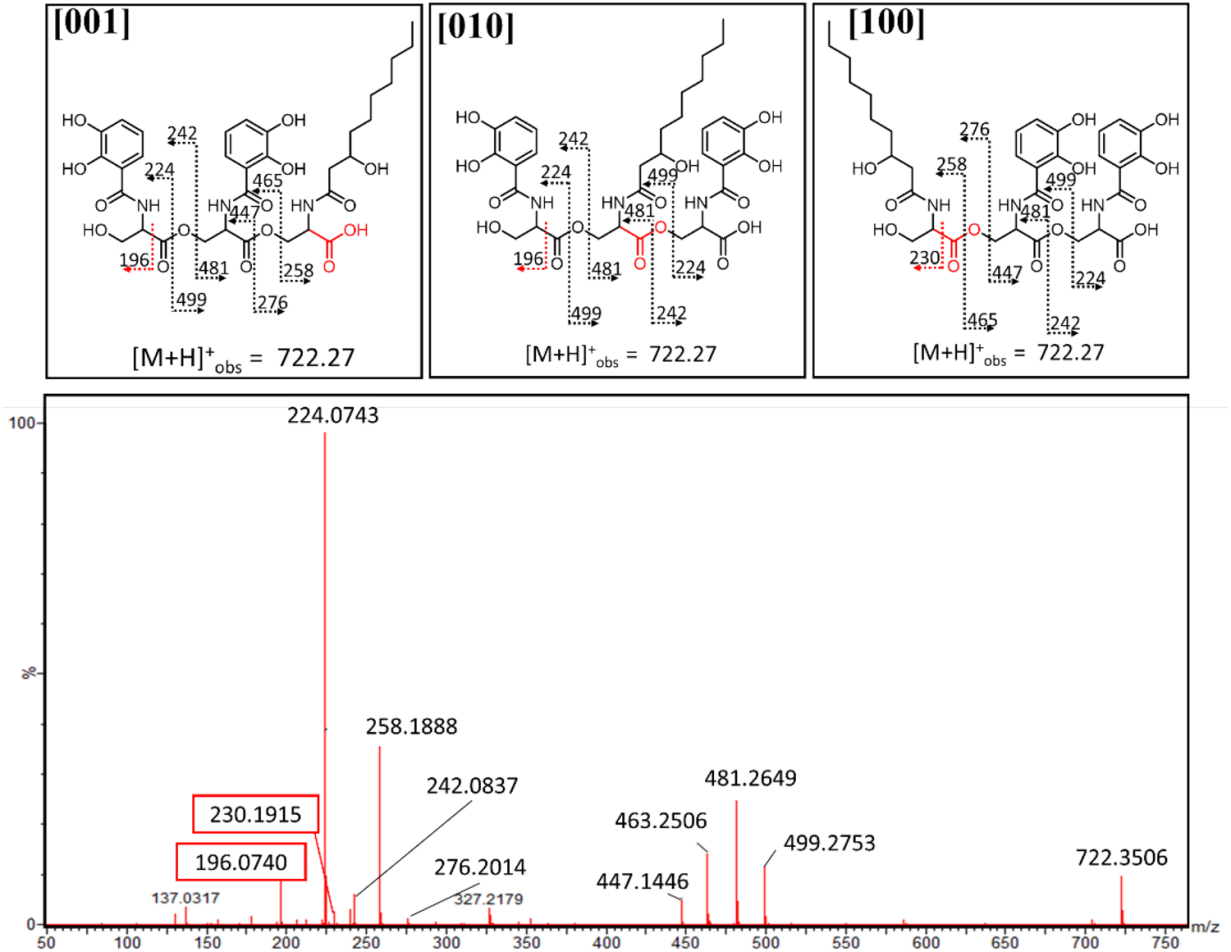
Tandem MS of *m*/*z* 722.27 for potential differentiation among the **[001]**, **[010]** and **[100]** isomers. A fragment ion of *m/z* 196 is consistent with premature release, while a fragment ion of *m/z* 230 is consistent with the hydrolysis of the amphi-enterobactin macrolactone

Distinguishing among the three **3-Ser-2-DHB-FA**^**C10:0−OH**^ structural isomers (Fig. 7) structures by tandem MS becomes more complex in comparison with the 2-Ser-1-DHB-FA structural isomer analysis. Premature release during biosynthesis would produce the 3-Ser-2-DHB-FA^C10:0−OH^ isomer **[001]**, whereas all three isomers, **[100]**, **[010],** and **[001],** would be produced from hydrolysis of the amphi-enterobactin macrolactone. The fragmentation at the N-terminal l-Ser is again the differentiating point among the isomers. Isomer [**100]** would result in a unique MS/MS fragment at *m/z* 230 [M + H]^+^. Unfortunately, both **[001]** and **[010]** isomers would produce a fragment with *m/z* 196 [M + H]^+^ in the same location, making these two isomers indistinguishable.

Tandem mass spectral analysis was carried out on the products with a protonated molecular mass of *m/z* 722.27 [M + H]^+^, consistent with **3-Ser-2-DHB-FA**^**C10:0−OH**^ (Fig. 7) and 750.38 [M + H]^+^, consistent with **3-Ser-2-DHB-FA**^**C12:0−OH**^ (Fig. S4). Tandem MS shows both of the fragments at *m/z* 196 and *m/z* 230. The *m/z* 230 ion confirms the presence of **[100]**, which can only arise from an amphi-enterobactin macrolactone hydrolysis product. The ion fragment at *m/z* 196 was identified by tandem MS and could arise from both **[001]** and **[010]**. These two products cannot be distinguished by tandem MS. However, a mixture of the 3-Ser-2-DHB-FA products is present in both the C10:0–OH and C12:0–OH compounds. The mixture of both fragments is evidence that amphi-enterobactin is produced and is hydrolyzed to [**100]**, and one of both of **[010]** and **[001]**, although the presence of all three isomers does not rule out the co-occurrence of premature release during biosynthesis.

## Conclusions

In summary, tandem MS analysis of the hydrolysis fragments of amphi-enterobactin in the culture supernatant establish that isomers **[10]** and **[100]** must arise from hydrolysis of the macrolactone amphi-enterobactin siderophore as opposed to prerelease of di-Ser or tri-Ser fragments during biosynthesis. Evidence for the **[10]** and **[100]** hydrolysis fragments is given by the unique MS/MS fragment at *m/z* 230 [M + H]^+^ arising from the C_10:0 OH_ derivatives (Figs. 5, 7) and *m/z* 258 [M + H]^+^ arising from the C12:0–OH derivatives (SI Figs. S3 and S4). These fragments establish amphi-enterobactin is fully formed and then hydrolyzed. Identification of products uniquely associated with prerelease, e.g., **[1]**, **[01]**, **[001]**, and **[0001],** is not possible since the fragments may also arise from hydrolysis of the amphi-enterobactin macrolactone.

Future experiments involving an in vitro analysis of the biosynthesis proteins for amphi-enterobactin could provide insight into the potential for premature release of incomplete fragments along the biosynthetic pathway for amphi-enterobactin. Previous results from reconstructing enterobactin synthetase activity reveal a pH dependence for the formation of enterobactin hydrolysis products [11]. At pH 7.5, enterobactin was the only product synthesized and released, while pH 8.8, the bis-catechol, bis l-Ser fragment, (DHB-l-Ser)_2_ was observed. This (DHB-l-Ser)_2_ intermediate was a result of premature release of the incompletely synthesized enterobactin from EntF at pH 8.8 rather than hydrolysis of enterobactin itself. As a control, when the synthesized enterobactin was incubated in pH 8.8 Tris–HCl buffer, enterobactin hydrolyzed to the DHB-Ser linear trimer, but not to the monomer or dimer. This study suggests that premature hydrolysis is pH dependent, and that at physiological pH, in vitro, no early release occurs [11].

*Campylobacter jejuni*, a bacterial strain that does not itself produce siderophores, contains a siderophore uptake system able to recognize and take up Fe(III)-siderophores produced by other bacterial species [12]. Further analysis of this uptake system identified the periplasmic binding protein, CeuE, involved in the uptake of Fe(III)-enterobactin showed a preference for binding to the Fe(III) complex of the tetradentate hydrolysis product of enterobactin, [Fe(III)-(DHB-l-Ser)_2_]^2−^, **[00]** [13]. The study rationalizes that utilizing the enterobactin hydrolysis products provides *C. jejuni* a competitive advantage because it avoids the metabolic costs associated with siderophore production. *C. jejuni* is able to recognize Fe(III)-enterobactin, but for the iron complex to enter the cytoplasm, the siderophore is hydrolyzed by the trilactone esterase Cee to form [Fe(III)-(DHB-l-Ser)_2_]^−^ [14].

During the NRPS-mediated biosynthesis, the release of the siderophore is catalyzed by the thioesterase domain either through hydrolysis, leading to a linear siderophore, or through an intramolecular nucleophilic attack, leading to the cyclized siderophore [15]. For hydrolysis to occur, water becomes the competing nucleophile and in turn releases a linear siderophore. The presence of linearized amphi-enterobactin has not yet been identified, but this does not eliminate the option that hydrolysis can still occur at any point during the biosynthesis.

Thus overall, only the **[01]**, **[001]** and **[0001]** the DHB-l-Ser fragments could originate from premature release during biosynthesis of amphi-enterobactin due to an inefficient biosynthetic pathway. The **[10]** and **[100]** fragments must arise from hydrolysis of the fully formed amphi-enterobactin macrolactone, which could occur enzymatically by a Fes-type esterase or non-enzymatically. The other possible fragment within the 3-Ser-3-DHB-FA series, **[010]**, is indistinguishable in the tandem MS analysis from the premature-release fragment; thus, without sufficient quantity of each fragment for NMR structural characterization, it is not possible to determine their origin.

If fragments are prematurely released during biosynthesis, it suggests that the NRPS pathway for amphi-enterobactin is inefficient and not dependable. Further investigations may shed light on the fidelity of the NRPS-catalyzed biosynthesis of amphi-enterobactin and the prevalence of incomplete synthesis of NRPS natural products.

## Supplementary Information

Below is the link to the electronic supplementary material.

Supplementary file1 (PDF 751 KB)
