# Supplementary material for: On the origin of amphi-enterobactin fragments produced by Vibrio campbellii species
Source: J Biol Inorg Chem. 2022 Jul 14;27(6):565–72. doi: 10.1007/s00775-022-01949-0 (PMC9470620; doi:10.1007/s00775-022-01949-0)
Supplement: Supplementary file 1 — Supplementary file1 (PDF 751 KB) [file 775_2022_1949_MOESM1_ESM.pdf]

Supplementary Information

On the origin of Amphi-enterobactin Fragments produced by *Vibrio campbellii* species

Aneta M. Jelowicki, Alison Butler

Department of Chemistry and Biochemistry, University of California, Santa Barbara, USA

Supplementary Information shows the possible hydrolysis fragments from amphi-enterobactin with a C12:0 OH fatty acid tail (Figure S1), and the tandem MS analysis of the 2-Ser-1-DHB-FA and 3-Ser-2-DHB-FA C12:0-OH isomer fragments from amphi-enterobactin with a C12:0-OH fatty acid tail (Figs. S2 and S3).

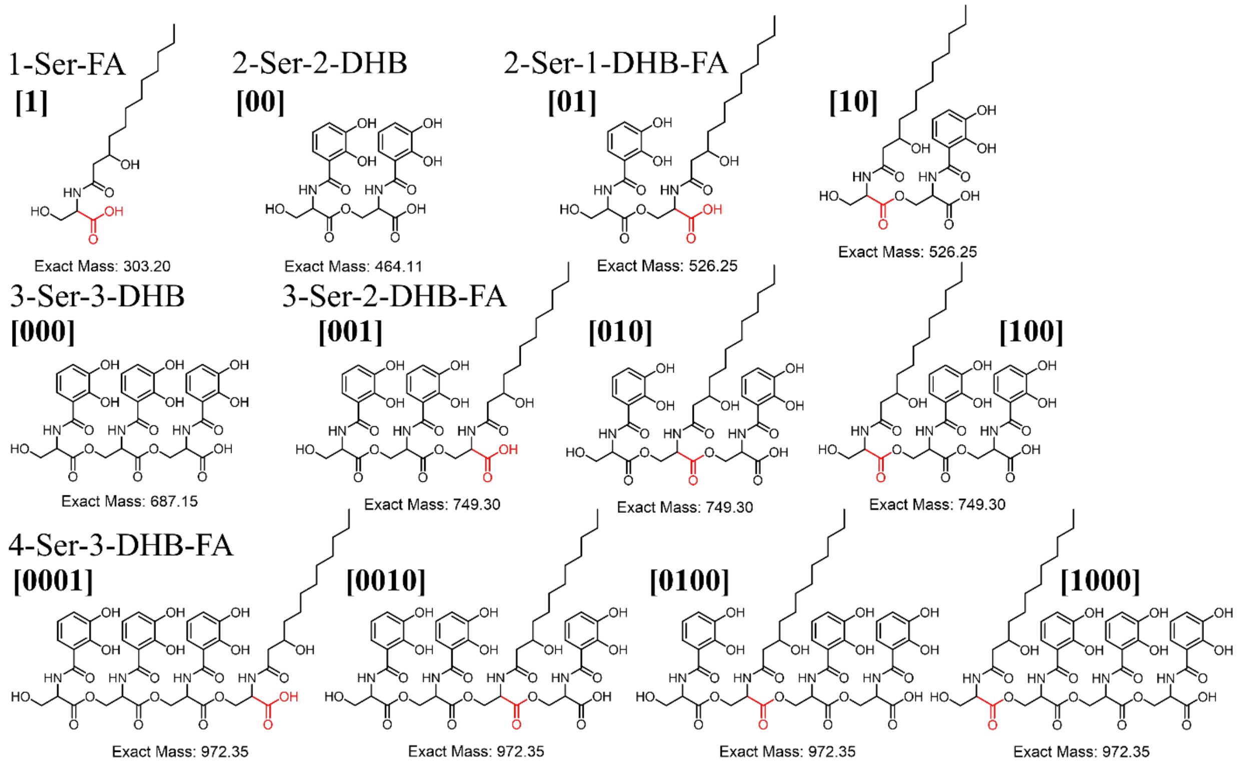

Fig. S1. The possible hydrolysis fragments from amphi-enterobactin with a C12:0 OH fatty acid tail. Compounds **[01], [001], [0001]** are the only structural possibilities for premature release. A mixture of compounds shown here, suggest breakdown by an esterase or by molecular hydrolysis. The carboxylate of L-Ser appended by the FA during biosynthesis is shown in red. This carboxyl would be tethered to the thioesterase domain.

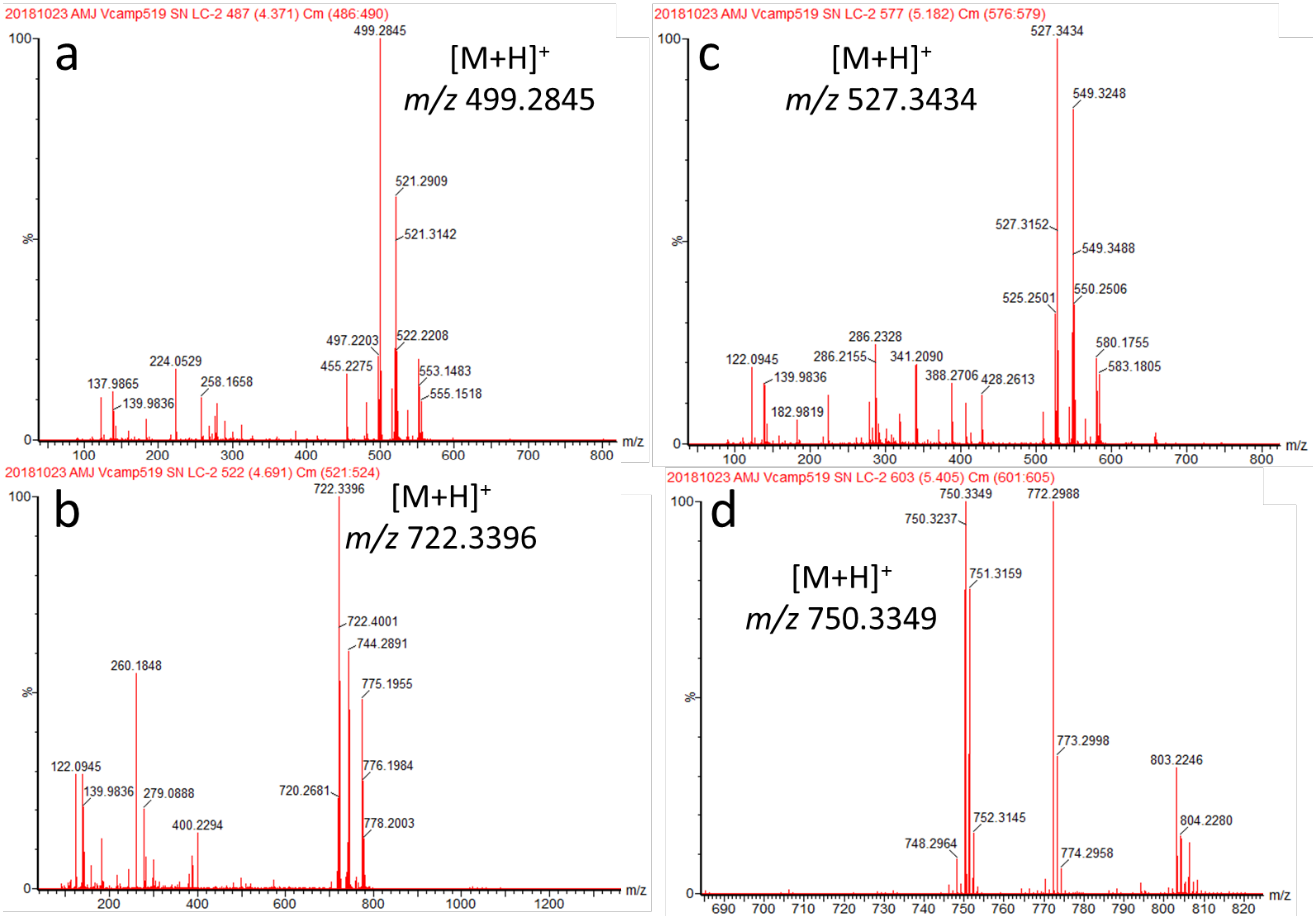

Fig. S2. MS spectra of Peaks A-D that correlate to masses of predicted amphi-enterobactin fragments, as seen in **Figure 1.**

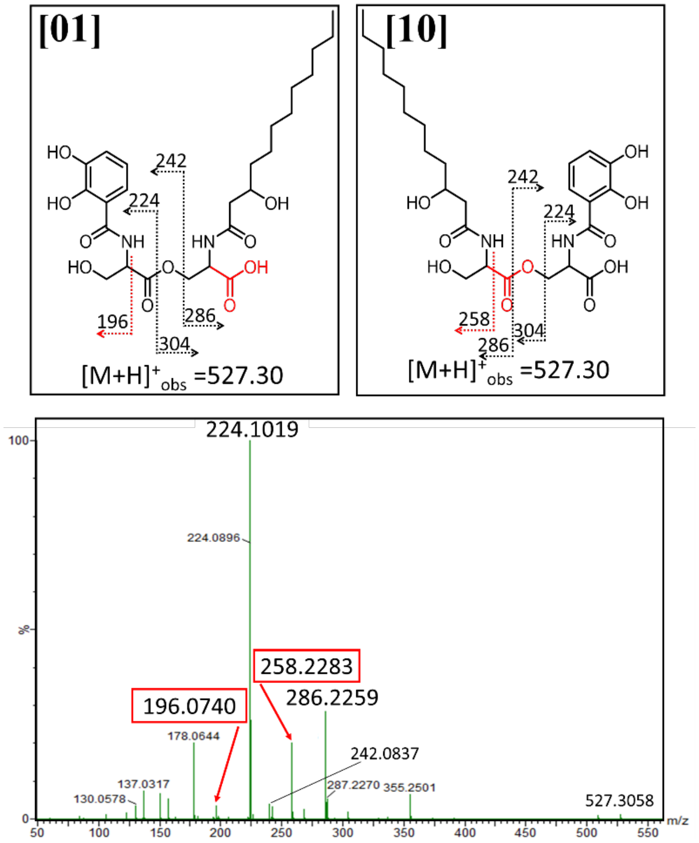

Fig. S3. Tandem MS of m/z 527.30 for differentiation between **[01]** and **[10].** A fragment ion of *m/z* 196 could result from premature release during biosynthesis or from hydrolysis of the amphi-enterobactin macrolactone, while a fragment ion of *m/z* 258 is consistent only with the hydrolysis of the amphi-enterobactin macrolactone.

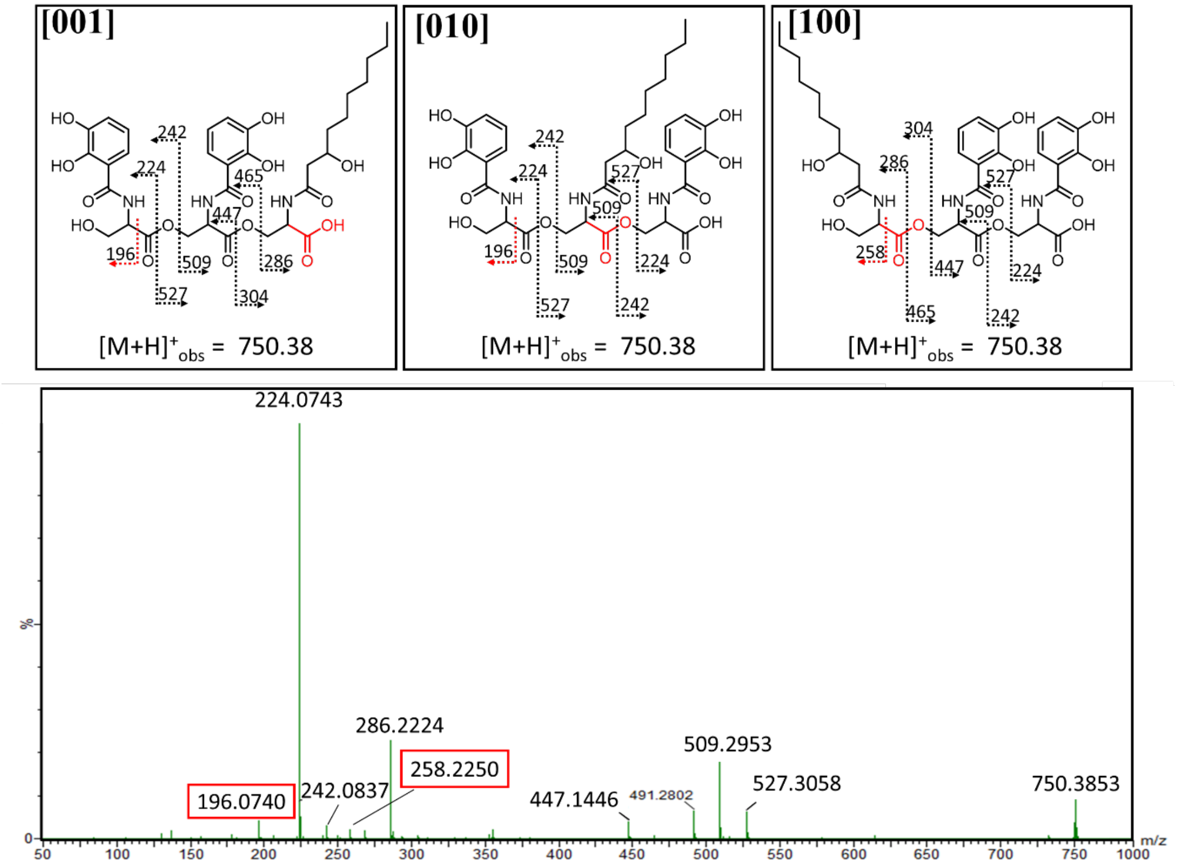

Fig. S4. Tandem MS of m/z 750.38 for potential differentiation among the **[001]**, **[010]** and **[100]** isomers. A fragment ion of *m/z* 196 could result from premature release during biosynthesis or from hydrolysis of the amphi-enterobactin macrolactone, while a fragment ion of *m/z* 258 is consistent only with the hydrolysis of the amphi-enterobactin macrolactone.
